# EP11 A case of COVID-19 induced myocarditis in a healthcare-worker

**DOI:** 10.1093/rap/rkaa052.010

**Published:** 2020-11-03

**Authors:** Shawki El-Ghazali, Sanjay Sivalokanathan, Michael Foley, Inithan Ganesaratnam, Simon Pearse, Amanda Varnava, Aneil Malhotra, Graham Cole, Taryn Youngstein

**Affiliations:** r1 Hammersmith Hospital, Imperial College NHS Trust, London, United Kingdom; r2 Kingston Hospital NHS trust, London, United Kingdom; r3 Wythenshawe Hospital and Manchester Royal Infirmary, Manchester, United Kingdom

## Abstract

**Case report - Introduction:**

The most recognised presentation of SARS-CoV-2 infection thus far has been respiratory, with hypoxia secondary to bilateral pneumonia and severe acute respiratory distress syndrome as a result of the host response to the virus.

We describe the investigation and management of a case of severe life threatening myocarditis, with no respiratory symptoms, occurring in a young healthcare-worker during the early pandemic.

**Case report - Case description:**

A 37-year-old male healthcare-worker presented with dizziness, fever, and diarrhoea. He had no previous medical history. A month prior he had experienced a 5-day fever which self-resolved.

On presentation, he was in cardiogenic shock with raised temperature. Blood tests demonstrated lymphocytopenia, raised troponin (peak 490ng/L) and BNP>35,000ng/L. He was transferred directly to critical care.

Repeated PCR-swab testing for SARS-CoV-2 were negative. All other viral-testing and autoimmune screen were negative. ECG showed sinus-rhythm and CT-chest demonstrated lower-lobe consolidation with pleural-effusions.

Echocardiogram revealed significantly impaired biventricular-function with LVEF of 21%. He had evidence of LV-wall thickening, bi-atrial dilation and raised pulmonary-artery systolic pressures. Despite IV antibiotics for pneumonia and shock-dose hydrocortisone 50mg QDS, he deteriorated requiring two inotropes. He was discussed with the ECMO-team at a tertiary-centre who suggested referral to our centre for consideration of immunosuppression and the patient was transferred.

Serology was sent for SARS-CoV-2 to GOSH on a research basis as at the time there was not commercially available or approved assay.

Cardiovascular Magnetic Resonance (CMR) was delayed due to inability to lie flat. After much discussion (Pre-RECOVERY trial results) 0.75mg/kg prednisolone was started with rapid effect. Inotropes were weaned within 24 hours with subsequent step-down to a cardiac ward. CMR-imaging confirmed myocarditis with characteristic tissue characterisation (raised T1/T2 mapping) and patchy late gadolinium-enhancement. Serial echocardiograms and serum cardiac biomarkers all improved in keeping with the clinical picture. The serological result returned and revealed the presence of the SARS-CoV-2 IgG.

ACE-inhibitors, beta-blockade and diuretics were utilised to manage secondary heart failure from myocarditis. He was discharged on weaning oral prednisolone with close follow-up from the rheumatology and cardiology teams. Blood tests 1-month post-discharge demonstrated normalisation of troponin and BNP. Repeat CMR demonstrated normalisation of LV function with no evidence of oedema or myocarditis, with only residual late gadolinium-enhancement.

**Case report - Discussion:**

This previously healthy young man presented during the initial peak of the COVID-19 pandemic and had considerable occupational exposure. At that point, although multiple confirmed cases of this condition had been identified, experience was still limited regarding extra-pulmonary manifestations.

Since that time our understanding of the variability of COVID-19 has become apparent. Literature describes a variety of presentations beyond pulmonary including cardiac, ENT, neurological and gastrointestinal, as well as systemic manifestation including hyperinflammatory states and vasculitis.

Myocarditis was identified at an early stage from imaging and blood tests, and this was presumed to be likely viral in origin. At that time, there were limited case reports of COVID-19 induced myocarditis, although this remained a differential once routine viral-screening returned negative. Repeated viral swabs for COVID-19 returned negative, however it was noted that he initially did have symptoms a month prior, and it was unclear whether his acute presentation was related to active COVID-19 infection or the sequelae of this. Serum antibody tests were sent to a specialist laboratory, and this ultimately confirmed previous COVID-19 exposure.

The decision was made to implement high-dose prednisolone therapy to manage what was felt to be a likely secondary inflammatory phenomenon of COVID-19 at a time when this was not really recognised. There was initial caution regarding this approach due to previous significant negative outcomes of steroid use in the SARS and MERS pandemics.

In the subsequent period after this gentleman’s case, the preliminary findings from the RECOVERY trial were made available, from which national guidance advises dexamethasone as the only current licensed therapy in managing COVID-19 in certain circumstances. Use and subsequent recovery following high-dose prednisolone suggests similar principles with this treatment, as well as managing cases who may present with non-pulmonary involvement of COVID-19.

**Case report - Key learning points:**

Our experience in this case suggests multiple factors from which we can learn from, although since the time of this gentleman’s presentation, a greater understanding of COVID-19 has already highlighted key areas of learning.

For this case, the recognition of possible myocarditis without respiratory symptoms as among the possible presentations of COVID-19 is notable. We were able to utilise different methods of imaging to assess the extent of cardiac inflammation and cardiac function. Of highlight would be through use of Cardiovascular Magnetic Resonance, which was implemented as part of the initial diagnostic process, as well as ongoing monitoring post-discharge. With this method we can identify subtle changes of the myocardium which can help detect early relapse, and in future may be of significance for diagnosis and monitoring of potential future COVID-19 associated myocarditis.

As we hope to one day move towards a post-COVID-19 global climate, perhaps the most important learning point is the possibility of delayed organ/systemic manifestation secondary to COVID-19 infection. For this case, the presumption is that he may have developed the primary infection 1 month prior to presentation. The context of repeated negative PCR-swab results, but the subsequent confirmation of prior exposure on antibody testing would potentially support this. His first presentation appeared to be a mild self-limiting illness, although his later acute presentation was that of significant potential risk, which fortunately he was able to recover from. This however highlights a particular concern regarding the long-term impact of COVID-19 not just on chronic co-morbidity such as long term cardiac failure or respiratory disease, but also the potential re-presentation of those who are deemed to have fully recovered from the virus. As time continues following the first wave of cases, we must remain vigilant of future complications yet to manifest.

